# Rumen Bacterial Diversity of 80 to 110-Day-Old Goats Using 16S rRNA Sequencing

**DOI:** 10.1371/journal.pone.0117811

**Published:** 2015-02-20

**Authors:** Xufeng Han, Yuxin Yang, Hailong Yan, Xiaolong Wang, Lei Qu, Yulin Chen

**Affiliations:** 1 College of Animal Science and Technology, Northwest A&F University, Yangling, Shaanxi, P. R. China; 2 College of Life Science, Yulin University, Yulin, Shaanxi, P. R. China; New England Biolabs, Inc., UNITED STATES

## Abstract

The ability of rumen microorganisms to use fibrous plant matter plays an important role in ruminant animals; however, little information about rumen colonization by microbial populations after weaning has been reported. In this study, high-throughput sequencing was used to investigate the establishment of this microbial population in 80 to 110-day-old goats. Illumina sequencing of goat rumen samples yielded 101,356,610 nucleotides that were assembled into 256,868 reads with an average read length of 394 nucleotides. Taxonomic analysis of metagenomic reads indicated that the predominant phyla were distinct at different growth stages. The phyla Firmicutes and Synergistetes were predominant in samples taken from 80 to 100-day-old goats, but Bacteroidetes and Firmicutes became the most abundant phyla in samples from 110-day-old animals. There was a remarkable variation in the microbial populations with age; Firmicutes and Synergistetes decreased after weaning, but Bacteroidetes and Proteobacteria increased from 80 to 110 day of age. These findings suggested that colonization of the rumen by microorganisms is related to their function in the rumen digestive system. These results give a better understanding of the role of rumen microbes and the establishment of the microbial population, which help to maintain the host’s health and improve animal performance.

## Introduction

The rumen is a complex ecosystem that harbors a wide variety of microorganisms, including bacteria, protozoa, archaea and fungi [1]. A principal function of the microbiome is the conversion of plant materials into digestible compounds that can be used by the animal host [2]. This function is of tremendous importance as it allows the conversion of solar energy stored in plant fibers into food products, such as milk and meat. Additionally, as the microbiome in the rumen undergoes long-term selection and evolution, the microbes and host form an interinhibitive and interdependent homeostatic relationship that has an important role in maintaining host health, improving performance, reducing environmental pollution, and ensuring food and animal product safety [3]. Therefore, studies of ruminal microbes represent a key area of nutrition research in ruminants, and it is important to improve our understanding of these complex microbial populations and their interactions.

Metagenomics is a new discipline that studies total microbial DNA extracted directly from the environment [4]. Therefore, using metagenomic sequencing technology, the gut microbiome can be immediately investigated and yields a large amount of raw data. Compared with 16S rRNA gene analysis techniques, using metagenomic sequencing technology to achieve high coverage can more accurately reflect the structure of the gut microbiome [5]. Metagenomic sequencing technology has been used to study the intestinal microbiome of different animal species and humans [6–10]. However, to date, no studies have reported the composition of the rumen microbial community in the Shaanbei white-cashmere (SBWC) goat.

The SBWC goat is the major cashmere and meat-producing animal raised in vast geographic areas in Northern Shaanxi province, P.R. China. Its cashmere is known as “soft gold,” and is a major economic resource for local farmers. However, the high mortality of lambs resulting from diarrheal disease has caused huge losses for farmers: more than 50% of weaned lambs are currently dying of diarrhea. Cho et al. [11] showed that diarrhea is closely related to the intestinal microbiome. Therefore, elucidating the composition of the gut bacterial community and its changes after weaning in these goats is essential to improve the health management and productivity of this important ruminant. To our knowledge, there has been no report of using sequencing techniques to study developmental changes in the ruminal microbial flora in this goat species after weaning. Thus, this study aimed to characterize the colonization process by ruminal microorganisms in goats from 80 to 110-day-old of age using high-throughput next-generation sequencing.

## Materials and Methods

### Animal handling and sampling

All experimental procedures with the goats used in this study received prior approval from the Experimental Animal Management Committee of Northwest A&F University. All surgery was performed under xylazine chlorhydrate anesthesia, and all efforts were made to minimize suffering.

Three goats that were 40 ± 1 day old with similar body weights were randomly chosen for installation of a rumen fistula. The goats were weaned when 60 days old and were fed a diet *ad libitum* twice daily at 08:30 and 18:00. The ingredients and nutrient composition of the diet are shown in S1 Table. Animals were maintained in a house with free access to water, and maintained their normal herd behavior.

We collected 50 mL of rumen contents when the goats were 80, 90, 100 and 110 day old. All collections were performed before the morning feeding (at 08:30). Rumen content samples were collected through the rumen fistula into a 50-mL plastic container. Rumen contents were squeezed through four layers of cheesecloth to remove particulate matter. The remaining ruminal liquid was stored at –80°C for DNA extraction.

### DNA extraction, PCR amplification of 16S rRNA and sequencing

Microbial genomic DNA was extracted from rumen samples using a stool DNA kit (OMEGA Bio-Tek, Norcross, GA, USA), according to the manufacturer’s instructions. The V4–V5 hypervariable regions of 16S rRNA were PCR amplified from microbial genomic DNA using the following universal primers: V515F, 5ʹ-GTGCCAGCMGCCGCGG-3ʹ; V907R, 5ʹ- CCGTCAATTCMTTTRAGTTT-3ʹ. PCR was carried out in triplicate 20-μL reactions containing 0.2 μM of each primer, 10 ng template DNA, 4 μL 5× FastPfu Buffer, 2 μL 2.5 mM dNTPs, and 0.4 μL FastPfu Polymerase (MBI Fermentas, Waltham, MA, USA). Thermocycling parameters were as follows: 2 min initial denaturation at 95°C; 30 cycles of denaturation at 95°C for 30 s, annealing at 55°C for 30 s, and elongation at 72°C for 30 s; and a final extension at 72°C for 5 min. PCR products were excised from 2% agarose gels and purified with the QIAquick Gel extraction kit (Qiagen, Venlo, The Netherlands). The DNA concentrations of the PCR products were determined using QuantiFluor-ST (Promega, U.S.) and amplicons from each reaction mixture were pooled at equimolar ratios based on the concentration of each amplicon. Barcoded V4 and V5 amplicons were sequenced using the pair-end method by Illumina Miseq with a six-cycle index read. The resulting sequences were then screened and filtered for quality and length. Sequences with a length shorter than 50 bp, having more than two primer mismatches, containing ambiguous characters or exhibiting a homopolymer run exceeding 6 bp, were removed [12].

### Bioinformatics and statistical analysis

The high-quality sequences were clustered into operational taxonomic units (OTUs) defined by 97% similarity. These OTUs were used for diversity (Shannon and Simpson), richness (Ace [13] and Chao [14]), and rarefaction curve analysis using MOTHUR (http://schloss.micro.umass.edu/) [15]. Taxonomic assignments of OTUs that reached the 97% similarity level were made using QIIME by comparison with the SILVA (http://www.arb-silva.de) [16], Greengene (http://rdp.cme.msu.edu/) [17] and RDP (http://greengenes.secondgenome.com/) [18] databases. A heat map was generated using the Heatmap 2 function of the “R gplots package” and genus information for the four groups [19]. We performed *t*-tests using the statistical analysis software SPSS version 19.0 for Windows (SPSS Inc., Chicago, IL, USA).

## Results

### Sequences

**Fig 1:**
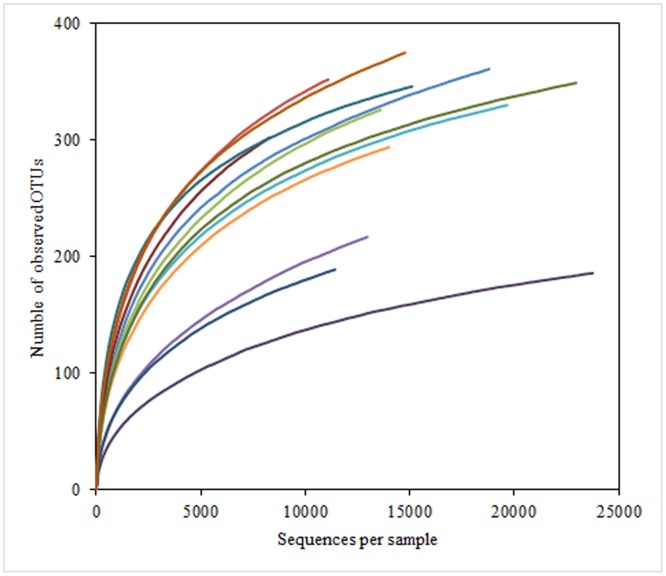
Rarefaction analysis of the different samples. Rarefaction curves of OTUs clustered at 97% sequence identity across different samples.

A total of 256,868 quality sequences were obtained from the 12 samples from three goats. These sequences included an average of 21,405 reads per rumen sample, and the average length of the quality sequences was 394 bp (Table 1). Most rarefaction curves for each sample approached the saturation plateau (Fig. 1), which indicated that the sampling effort had sufficient sequence coverage to accurately describe the bacterial composition of each group. Indices of bacterial richness based on OTUs were estimated by the method of Ace and Chao, and indices of bacterial diversity were determined using the method of Simpson and Shannon (Table 1). Among the 12 samples, the total number of OTUs detected by our analysis was 3627, with an average of 302 OTUs per sample (Table 1).

**Table 1:** Diversity estimation of the 16S rRNA gene libraries of the rumen of goats from the sequencing analysis^a^.

Sample ID^b^	Reads	OTU	Ace	Chao	Shannon	Simpson
**21**	18794	361	453 (422, 500)	456 (418, 520)	3.2 (3.17, 3.23)	0.1378 (0.1338, 0.1419)
**22**	11098	352	446 (415, 493)	447 (409, 511)	3.66 (3.62, 3.69)	0.0952 (0.0908, 0.0996)
**23**	13598	326	419 (387, 467)	417 (380, 480)	3.18 (3.15, 3.21)	0.1208 (0.1172, 0.1244)
**31**	12964	217	448 (392, 521)	345 (290, 441)	2.65 (2.62, 2.68)	0.1665 (0.1612, 0.1718)
**32**	19651	330	401 (376, 441)	404 (372, 459)	2.83 (2.8, 2.87)	0.2207 (0.2148, 0.2265)
**33**	14007	294	371 (343, 414)	377 (341, 439)	3.38 (3.35, 3.41)	0.0773 (0.0751, 0.0795)
**41**	11418	189	313 (277, 365)	266 (230, 333)	2.42 (2.38, 2.45)	0.2062 (0.2001, 0.2123)
**42**	8272	302	406 (371, 458)	391 (355, 453)	3.24 (3.2, 3.29)	0.1339 (0.128, 0.1397)
**43**	22946	349	415 (392, 453)	426 (393, 484)	3.44 (3.42, 3.46)	0.0704 (0.0689, 0.072)
**51**	23747	186	247 (222, 290)	254 (221, 318)	1.95 (1.93, 1.97)	0.2812 (0.2765, 0.2859)
**52**	15106	346	409 (386, 447)	447 (402, 529)	4.05 (4.02, 4.08)	0.0425 (0.0412, 0.0438)
**53**	14768	375	476 (442, 526)	516 (459, 611)	3.8 (3.77, 3.83)	0.0771 (0.074, 0.0803)

^a^ The operational taxonomic units (OTUs) were defined with 3% dissimilarity. The richness estimators (ACE and Chao) and diversity indices (Shannon and Simpson) were calculated.

^b^ The 80-day-old group samples included goats 21, 22 and 23; the 90-day-old group samples included goats 31, 32 and 33; the 100-day-old group samples included goats 41, 42 and 43; the 110-day-old group samples included goats 51, 52 and 53.

### Taxonomic composition

**Fig 2:**
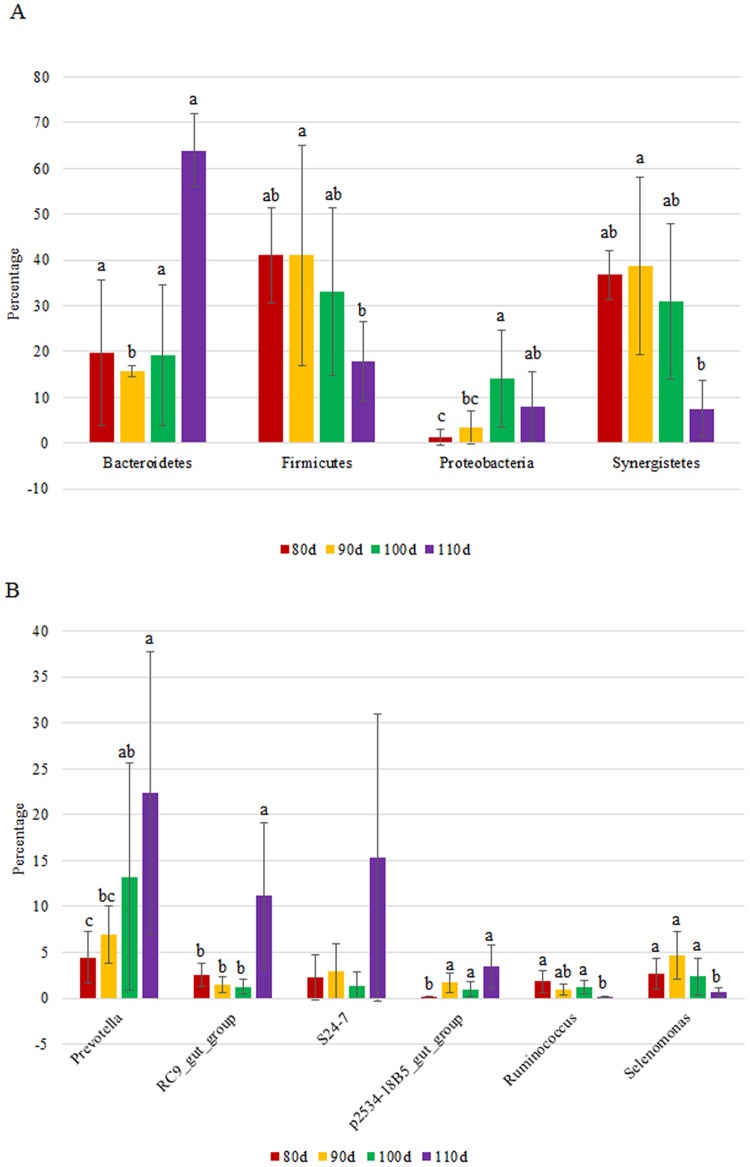
Temporal changes in the relative abundance (% reads) of (A) the most dominant phylum and (B) the most dominant genera in the rumen microbial community of goats with age. Error bars represent the SD of three samples. Boxes with a different letter above the error bars are significantly different at P<0.05 or P<0.1 (Firmicutes) by *t*-test analyses.

Based on the SILVA taxonomic database and using the analysis program QIIME [20], all sequences were classified from phylum to species. Twenty-five different phyla were detected in these samples. The four groups showed very dissimilar taxonomic compositions, even at the phylum-level distributions (Fig. 2 and S2 Table). Within the 80-day-old group, Firmicutes (41.01%), Synergistete (36.76%) and Bacteroidetes (19.70%) were the dominant phyla. The 90-day-old group was also dominated by Firmicutes, Synergistete and Bacteroidetes, representing 41.02%, 38.78% and 15.63% of the total reads, respectively. In the 100-day-old group, Firmicutes, Synergistetes, Bacteroidetes and Proteobacteria were the most common groups and accounted for 33.11%, 30.99%, 19.12% and 13.96% of the reads, respectively. Finally, the 110-day-old group was dominated by Bacteroidetes, Firmicutes, Proteobacteria and Synergistetes, which represented 63.96%, 17.92%, 8.05% and 7.50% of the reads, respectively.

**Fig 3:**
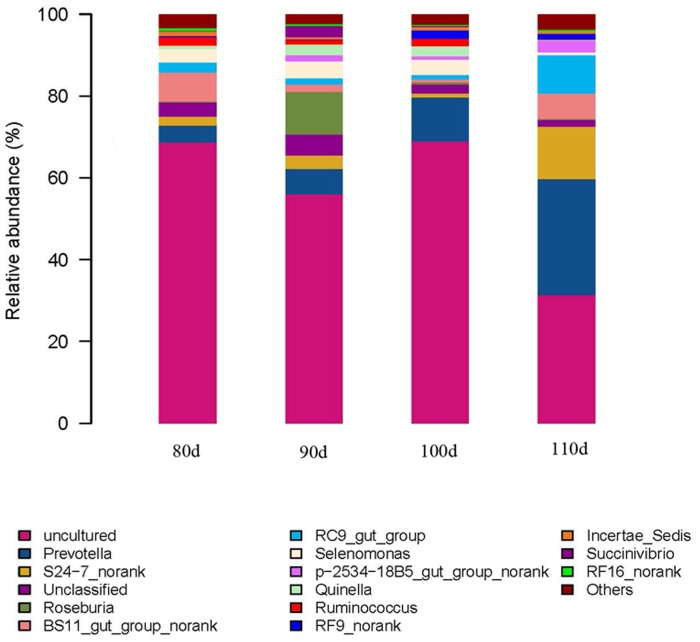
Genera-level composition of the rumen microbiome. A color-coded bar plot showing the average bacterial genera distribution across the different age groups that were sampled.

At the genus level, the sequences could be assigned to 82 different genera. The most abundant genera (the relative abundance of genera representing more than 1% of the four libraries) among the libraries were determined to yield insights into which bacteria might be the most important (Fig. 3 and S3 Table). In the 80-day-old group, the *BS11_gut_group* was predominant with an abundance of 7.45%, followed by *Prevotella*, *Selenomonas*, *RC9_gut_group*, *S24–7* and *Ruminococcaceae*. In the 90-day-old group, the most abundant genera were *Roseburia*, *Prevotella*, *Selenomonas*, *Succinivibrio*, *Quinella*, *S24–7*, p*2534–18B5_gut_group*, *BS11_gut_group* and *RC9_gut_group*, which together accounted for 68.90% of the total sequences. In the 100-day-old group, the most abundant sequences were those related to *Prevotella*, *Selenomonas*, *RF9*, *Quinella*, *S24–7*, *RC9_gut_group*, *Incertae_Sedis*, *Ruminococcus* and *p2534–18B5_gut_group*. For the 110-day-old group, it was numerically dominated by sequences related to *Prevotella*, *S24–7*, *RC9_gut_group*, *BS11_gut_group*, *p2534–18B5_gut_group* and *RF9*. We also noticed that there were many unclassified and uncultured bacteria in the samples from the 80-, 90-, 100- and 110-day-old groups, representing 72.14%, 57.59%, 69.67% and 32.82% of the sequences, respectively.

**Fig 4:**
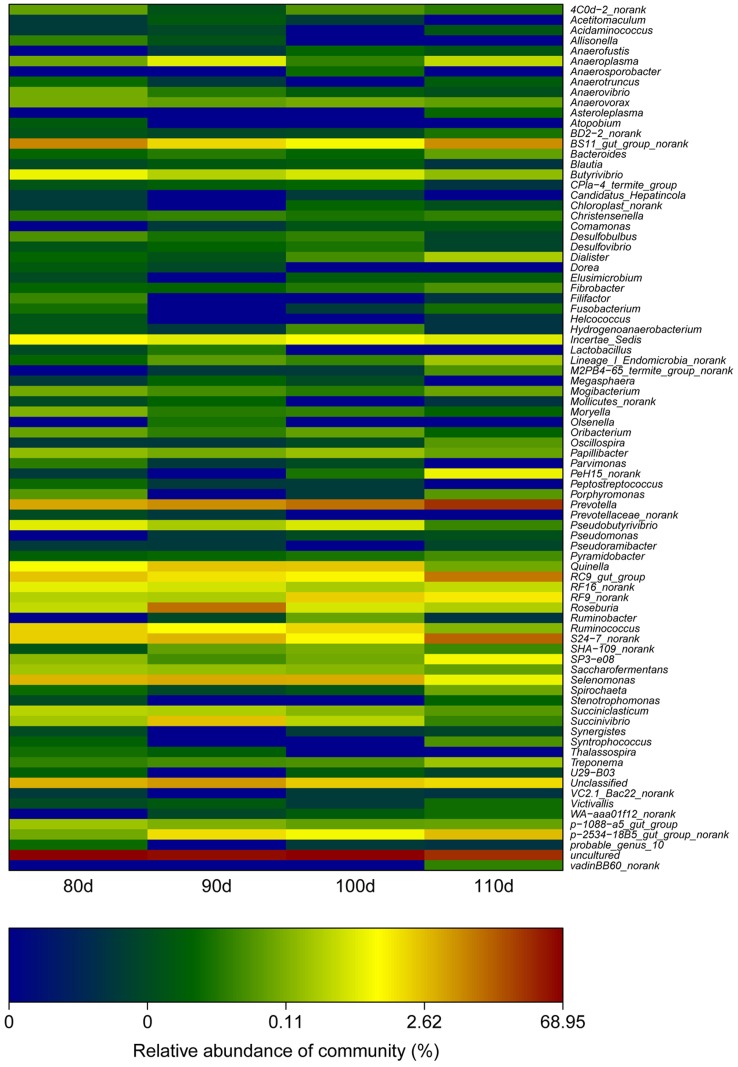
A heat map of the rumen microbiome composition at the species level. The heat map indicates the relative percentage of each genera for the different age groups sampled.

In the rumen, there are four primary types of cellulose-degrading bacteria, *Ruminococcus flavefaciens*, *Fibrobacter succinogenes*, *Butyrivibrio fibrisolvens* and *Clostridium sp*. In this study, all four types of bacteria were detected in the 110-day-old group (Fig. 4 and Table 2). In the other groups, only three types of cellulose-degrading bacteria were detected, *Ruminococcus flavefaciens*, *Fibrobacter succinogenes* and *Butyrivibrio fibrisolvens*, but not *Clostridium* sp. However, these cellulose-degrading bacteria were present at a low overall relative abundance (<1% of the total microbial community).

**Table 2:** The relative abundance (%) of cellulose-degrading bacteria in different age groups of goats.

**Species**	**80 d**	**90 d**	**100 d**	**110 d**
***Butyrivibrio_fibrisolvens***	0.06	0.04	0.12	0.02
***Fibrobacter_succinogenes***	0.01	0.01	0.02	0.03
***Ruminococcus_flavefaciens***	0.27	0.08	0.50	0.04
***Clostridium*_sp**	0.00	0.00	0.00	0.01

### The effect of age on the relative abundance and diversity of bacterial communities

With age, we observed a profound change in rumen microbial composition at the phylum and genus levels (Fig. 2). The age of the goats showed a statistically significant effect on the relative abundance of Bacteroidetes (P<0.05 by *t*-test), which increased with age and became the most abundant phylum in samples from the 110-day-old group. By contrast, the Firmicutes significantly decreased with age and were less abundant (P<0.1) in samples from the 110-day-old group compared with the 90-day-old group. The Synergistetes were significantly reduced (P<0.05) in the 110-day-old group. Finally, the Proteobacteria showed a trend of increasing with age: the relative abundance of Proteobacteria in the 100-day-old group was significantly higher than that in the 80-day-old group (P<0.05).

In this study, we found that the relative abundances of genera were also affected by age (the six most abundant genera were analyzed and are shown in Fig. 2). With age, the relative abundances of *Prevotella*, *RC9_gut_group* and *p2534–18B5_gut_group* significantly increased; however, the relative abundance of *Ruminococcus* and *Selenomonas* significantly declined (P<0.05). The genera *S24–7* showed an increasing trend; however, the difference among the four groups was not statistically significant. Among these genera, *Prevotella* showed a linear increase in abundance with age, reaching a maximum in the 110-day-old group. With age, the relative abundance of unclassified and uncultured bacteria significantly decreased (P<0.05), and the relative abundance of unclassified and uncultured bacteria in the 110-day-old group was significantly lower than that in the 90-day-old group (S3 Table).

## Discussion

In prokaryotes, the 16S ribosomal RNA (rRNA) is a small subunit of an rRNA comprising a ribosome and has a length of ~1500 bp. The underlying sequence diversity among different bacterial species [21–23] have meant that 16S ribosomal RNA gene sequences have been used widely for phylogenetic studies and taxonomic classification [24, 25]. Here, we used next-generation sequencing technology to characterize changes in the goat rumen bacterial community from 80 to 110 day of age, and to determine how the composition changes during normal development.

Many studies have shown that Bacteroidetes and Firmicutes are numerically the most dominant phyla in the gut microbiome of terrestrial mammals [6, 26–28]. However, in our data, the Firmicutes and Synergistetes were the most abundant in samples obtained from 80 to 100-day-old goats, while the microbial community of the 110-day-old goats was dominated by Bacteroidetes and Firmicutes. This difference suggested that the rumen microbial composition of lambs is different from that of adult goats. The rumen microbial composition of 110-day-old goats showed a convergence towards a mature bacterial composition [29]. This finding was consistent with a previous study by Jami *et al*. [29] in which the phylum Bacteroidetes was significantly less abundant in samples from the 1–3-day-old animal group compared with all other groups, but became the most abundant phylum in samples from older animals.

With age, the four most abundant phyla in the microbial community showed typical changes. The phyla Bacteroidetes and Proteobacteria tended to increase, but the phyla Firmicutes and Synergistetes tended to decrease. The effect of age on gut bacterial communities was also reported by Koenig *et al*. [30] and Yatsunenko *et al*. [31]. These studies reported age-related changes in the human gut microbiome. The reason for this result might be related to the function of bacteria. After weaning, the digestion of food content-associated bacteria gradually replaces bacteria that were established during breast-milk feeding in the rumen of goats. Morrill et al. [32] reported that by keeping the indigenous intestinal microbiome in balance, the animal is ready to respond successfully to eventual pathogen colonization. When a microbiome imbalance occurs, probiotics populations may diminish and pathogen microorganisms may increase, causing animal diarrhea [33]. In our study, the dramatic changes in the bacterial microbiome may explain why lambs frequently develop diarrhea. The dramatic changes in microbiome suggested that it had not yet reached a stable, balanced state. The microbiome is an extremely fragile ecosystem, and changes in the external environment (e.g. drastic weather changes, changes in feed components and conditions after transport), can cause diarrhea in kids.

In this study, pronounced differences in the microbiota composition among different age groups were evident. The phylum Bacteroidetes in the 110-day-old group was significantly higher than in the 90-day-old group, whereas the Firmicutes in the 110-day-old group was significantly lower than in the 90-day-old group, which is consistent with previous studies [29, 34]. Similarly, De Filippo *et al*. [35] reported that a much higher proportion of Bacteroidetes and a lower proportion of Firmicutes could be observed in African children compared with European children. These differences were a consequence of the different diets consumed by these children; the African diet is mainly composed of plant fiber, whereas the European diet is high in animal protein, sugar, starch and fat [35]. The goats studied here undergo dietary changes similar to the above study. After weaning, the goat diet is mainly feed rather than milk, which is of high caloric value, and is rich in protein, fat and sugar [36]. In the current study, the phylum Proteobacteria in the 100-day-old group was significantly higher than in the 80 and 90-day-old groups. This finding is inconsistent with above studies [29, 34], in which the relative abundance of Proteobacteria significantly decreased with age. This difference may be because of species-specific differences.

Surprisingly, the phylum Synergistetes, which is known for its ability to degrade amino acids and pyruvate, was the most abundant in our study. To date, no study has reported Synergistetes to be the dominant microbe in the rumen microbiome. This finding indicated that Synergistetes may be unique to SBWC goats, and could play a central role in ruminal digestion of breast milk. However, to thoroughly understand the changes in the abundance of this bacteria, additional studies will be needed.

This study also detected some age-related changes in the population structure at the genus level. Among the different genera represented, *Prevotella* was the most abundant in the adult rumen. Previous studies, using culture methods, showed that *Prevotella* strains comprised more than 50% of the total bacteria from the rumens of goats [37]. A quantitative study showed that *Prevotella* accounted for 42–60% of the total bacteria in the rumen [38], and 16S rRNA gene pyrosequencing indicated that *Prevotella* was the most abundant bacterial genus, accounting for an average of 52% of all reads in the bovine rumen microbiome [39]. Here, we found that *Prevotella* tended to increase with age, and became the most predominant ruminal genus in the 110-day-old group (accounting for 22.37% of all reads), which was consistent with previous studies. Additionally, this observation concurs with a recent study by Jami *et al*. [29], in which the genus *Prevotella* became the dominant genera in the bovine rumen when high-fiber diets were introduced. Members of the *Prevotella* genus have important roles in the utilization of feed proteins within the rumen microbial ecosystem [40], which could explain the age-related changes in *Prevotella* that we observed.

Cellulose-degrading bacteria have been extensively studied in recent decades because of their important role in supporting the host [41]. These studies have determined that the dominant cellulose-degrading bacteria are dissimilar in various animals. *Bacillus*, *Vibrio*, *Aeromonas* and *Enterobacter* are the main cellulose-degrading bacterial genera in grass carp intestines [42, 43], whereas *Ruminococcus* spp. and *Fibrobacter* spp. are the main cellulose-degrading bacterial genera in the rumen [44, 45]. We detected *Ruminococcus flavefaciens*, *Fibrobacter succinogenes*, *Butyrivibrio fibrisolvens* and *Clostridium* sp could be detected; however, their relative abundances were low (no more than 0.50% of all reads). Our results are consistent with those of Zened *et al*. [46], who found that *Fibrobacter* and *Ruminoccocus* represented 0.34% and 0.92% of the total bacterial community in animals fed a LS diet, respectively. Furthermore, de Menezes *et al*. [47] reported that *Fibrobacteres* were considerably more prevalent in the solid phase than in the liquid phase, which could in part explain the low relative abundances of cellulose-degrading bacteria detected here. Yang *et al*. [48] found that *R. flavefaciens* abundance was 100 times greater than that of *R. albus* in the rumen, which could explain the absence of *R. albus* in our study.

Notably, we detected a large number of bacteria in the rumen of goats that belonged to unclassified and uncultured genera, based on the current 16S RNA gene sequence databases. This finding suggested that SBWC goats might possess a specific intestinal microbiome and reflected the fact that few studies of this type of goat have been previously conducted. Additionally, the relative abundances of unclassified and uncultured bacteria significantly reduced with age, which indicated that the bacterial microbiome of adult goats was better classified compared with the microbiome of young goats. To better characterize these unknown bacteria and their special roles in the hosts, further studies are required.

In conclusion, our study based on 16S rRNA gene sequencing reports the overall composition of the bacterial communities in the goat rumen ecosystem from 80 to 110 days of age. We revealed that age has significant effects on the microbial community in the rumen of goats, and the dominant genera and specific composition of the microbial community change with age. These observations provided a better understanding of how the bacterial ecosystem develops in goats after weaning.

## Supporting Information

S1 Table Ingredients and chemical composition of diets. ^a^ Supplies per kg of diet: 99.2 mg Mn, 50 mg Fe, 84.7 mg Zn, 10 mg Cu, 1 mg I, and 0.2 mg Se. ^b^ Supplies per kg of diet: 9000 IU vitamin A, 2000 IU vitamin D, and 18 IU vitamin E.
(DOCX)

S2 Table The relative abundance (%) of bacterial groups (phylum level) in different age groups of goats. (DOCX)

S3 Table The relative abundance (%) of predominant genera (more than 1% of total reads in the corresponding group) in different age groups of goats. (DOCX)
