# Supplementary material for: Rumen Bacterial Diversity of 80 to 110-Day-Old Goats Using 16S rRNA Sequencing
Source: PLoS One. 2015 Feb 20;10(2):e0117811. doi: 10.1371/journal.pone.0117811 (PMC4336330; doi:10.1371/journal.pone.0117811)
Supplement: S1 Table — a Supplies per kg of diet: 99.2 mg Mn, 50 mg Fe, 84.7 mg Zn, 10 mg Cu, 1 mg I, and 0.2 mg Se. b Supplies per kg of diet: 9000 IU vitamin A, 2000 IU vitamin D, and 18 IU vitamin E. (DOCX) [file pone.0117811.s001.docx]

| **Ingredient (% dry matter)** |  |
| --- | --- |
| **Alfalfa hay** | 40.00 |
| **Corn** | 38.50 |
| **Soybean meal** | 15.00 |
| **Wheat bran** | 5.00 |
| **Corn gluten meal** | 17.0 |
| **Peanut meal** | 6.50 |
| **Dicalcium phosphate** | 0.25 |
| **Limestone** | 0.25 |
| **Salt** | 0.50 |
| **Mineral mix^a^** | 0.25 |
| **Vitamin mix^b^** | 0.25 |
| **Calculated composition** | |
| **Metabolizable energy, MJ/kg** | 11.91 |
| **Crude protein (% dry matter)** | 16.30 |
| **Crude fiber (% dry matter)** | 15.91 |
| **Calcium (% dry matter)** | 0.87 |
| **Phosphorus (% dry matter)** | 0.37 |
