# Supplementary material for: Rumen Bacterial Diversity of 80 to 110-Day-Old Goats Using 16S rRNA Sequencing
Source: PLoS One. 2015 Feb 20;10(2):e0117811. doi: 10.1371/journal.pone.0117811 (PMC4336330; doi:10.1371/journal.pone.0117811)
Supplement: S2 Table — (DOCX) [file pone.0117811.s002.docx]

| **Phylum** | **80 d** | **90 d** | **100 d** | **110 d** |
| --- | --- | --- | --- | --- |
| **Actinobacteria** | 0.01 | 0.02 | 0.00 | 0.00 |
| **Bacteroidetes** | 19.70 | 15.63 | 19.12 | 63.96 |
| **Chlamydiae** | 0.00 | 0.00 | 0.02 | 0.02 |
| **Chloroflexi** | 0.05 | 0.01 | 0.00 | 0.01 |
| **Cyanobacteria** | 0.07 | 0.08 | 0.21 | 0.08 |
| **Elusimicrobia** | 0.02 | 0.06 | 0.06 | 0.24 |
| **Fibrobacteres** | 0.02 | 0.01 | 0.03 | 0.05 |
| **Firmicutes** | 41.01 | 41.02 | 33.11 | 17.92 |
| **Fusobacteria** | 0.02 | 0.00 | 0.00 | 0.02 |
| **Lentisphaerae** | 0.00 | 0.01 | 0.00 | 0.02 |
| **Planctomycetes** | 0.24 | 0.14 | 0.12 | 0.09 |
| **Proteobacteria** | 1.25 | 3.34 | 13.96 | 8.05 |
| **Spirochaetae** | 0.05 | 0.05 | 0.08 | 0.29 |
| **Synergistetes** | 36.76 | 38.78 | 30.99 | 7.50 |
| **Tenericutes** | 0.36 | 0.71 | 1.93 | 1.39 |
| **Unclassified** | 0.44 | 0.12 | 0.37 | 0.36 |
