# Supplementary material for: Rumen Bacterial Diversity of 80 to 110-Day-Old Goats Using 16S rRNA Sequencing
Source: PLoS One. 2015 Feb 20;10(2):e0117811. doi: 10.1371/journal.pone.0117811 (PMC4336330; doi:10.1371/journal.pone.0117811)
Supplement: S3 Table — (DOCX) [file pone.0117811.s003.docx]

| **Genera** | **80 d** | **90 d** | **100 d** | **110 d** |
| --- | --- | --- | --- | --- |
| ***Anaeroplasma*** | 0.09 | 0.49 | 0.03 | 0.34 |
| ***BS11_gut_group*** | 7.45 | 1.52 | 0.90 | 7.29 |
| ***Butyrivibrio*** | 0.66 | 0.28 | 0.63 | 0.15 |
| ***Incertae_sedis*** | 0.94 | 0.49 | 1.26 | 0.54 |
| ***Prevotella*** | 4.46 | 6.98 | 13.20 | 22.37 |
| ***Pseudobutyrivibrio*** | 0.49 | 0.23 | 0.48 | 0.04 |
| ***Quinella*** | 0.66 | 3.04 | 1.69 | 0.11 |
| ***RC9_gut_group*** | 2.51 | 1.47 | 1.27 | 11.23 |
| ***RF16*** | 0.61 | 0.50 | 0.22 | 0.42 |
| ***RF9*** | 0.26 | 0.21 | 1.89 | 1.05 |
| ***Roseburia*** | 0.34 | 12.54 | 0.53 | 0.26 |
| ***Ruminococcus*** | 1.85 | 0.91 | 1.22 | 0.13 |
| ***S24-7*** | 2.27 | 2.93 | 1.40 | 15.32 |
| ***SP3-e08*** | 0.15 | 0.04 | 0.13 | 0.92 |
| ***Saccharofermentans*** | 0.23 | 0.14 | 0.10 | 0.08 |
| ***Selenomonas*** | 2.71 | 4.68 | 2.40 | 0.72 |
| ***Succiniclasticum*** | 0.32 | 0.23 | 0.18 | 0.06 |
| ***Succinivibrio*** | 0.20 | 3.17 | 0.37 | 0.03 |
| ***Treponema*** | 0.03 | 0.05 | 0.07 | 0.21 |
| ***p-1088-a5_gut_group*** | 0.22 | 0.13 | 0.11 | 0.08 |
| ***p2534-18B5_gut_group*** | 0.10 | 1.68 | 1.01 | 3.47 |
| **Unclassified** | 3.42 | 4.28 | 1.82 | 1.88 |
| **uncultured** | 68.72 | 53.31 | 67.85 | 30.94 |
